# Extraglandular ocular involvement and morbidity and mortality in primary Sjögren’s Syndrome

**DOI:** 10.1371/journal.pone.0239769

**Published:** 2020-09-25

**Authors:** Priya M. Mathews, Susan A. Robinson, Anisa Gire, Alan N. Baer, Esen K. Akpek

**Affiliations:** 1 Ocular Surface Disease Clinic, Wilmer Eye Institute, Johns Hopkins University School of Medicine, Baltimore, Maryland, United States of America; 2 Division of Rheumatology, Department of Medicine, Johns Hopkins University School of Medicine, Baltimore, Maryland, United States of America; National Institute of Dental and Craniofacial Research, UNITED STATES

## Abstract

**Purpose:**

To report the significance of extraglandular ocular involvement and long-term systemic morbidity and mortality in primary Sjögren’s Syndrome (SS).

**Methods:**

This retrospective, longitudinal cohort study included consecutive patients with primary SS evaluated at a tertiary referral center. An electronic chart review was performed and all available data were extracted from clinic visits between October 1999 and March 2019. The primary outcome measures included occurrence of extraglandular ocular manifestations of SS, serological markers, prevalence of malignancy, and incidence of death.

**Results:**

One hundred and twenty-six SS patients with minimum 3 years of follow-up (median 9.6, range 3.0–15.9 years, total of 1,235 patient-years) were included. Of those, 10 patients with inflammatory keratolysis or scleritis had 2.3 times greater likelihood of death compared to the rest of the cohort (OR = 2.3, 95% confidence interval [CI] 0.5 to 4.0, p = 0.01) due to SS related complications. The lifetime prevalence of *any* malignancy in the entire cohort was 15.5%. The most common hematologic malignancy was non-Hodgkin’s lymphoma (4.8%) and the most common solid malignancy was breast cancer (6.0%). Men SS patients were more likely to have a history of or concurrent malignancy compared to women (30.0% versus 13.7%, p = 0.16) and double the mortality (OR = 2.1, 95% CI 0.09 to 1.4, p = 0.04), independent of malignancy.

**Conclusions:**

SS patients with serious ocular manifestations, particularly men, may be at greater risk for mortality due to SS complications. The eye seems to be the barometer of systemic disease activity.

## Introduction

Sjögren’s Syndrome (SS) is a chronic, debilitating autoimmune disease with a prevalence of approximately 43 cases per 100,000 individuals worldwide [1,2]. The disease is characterized by exocrine glandular damage leading to dry eye and dry mouth, but also has direct effects on extraglandular organs in 20–60% of individuals [3–7]. There is a wide spectrum of disease severity, ranging from mild sicca symptoms to severe multi-organ manifestations. Quantitative assessments have been developed to measure disease severity and activity particularly for clinical research, such as the European League Against Rheumatism (EULAR) Sjögren’s Syndrome Patient Reported Index (ESSPRI) and Sjögren’s Syndrome Disease Activity Index (ESSDAI) [8]. Regrettably, these instruments do not appropriately include extraglandular ocular manifestations or dry eye when assessing disease severity or activity [9].

Recent studies have elucidated that patients with extraglandular manifestations of SS particularly vasculitis or lymphoma are at higher risk for mortality [7,10]. Also, men with primary SS may have up to three times higher mortality rate compared to women [11–13], which was attributed to sex differences in the pathophysiology of the disease [14]. However, we noted a delay in the diagnosis of men patients largely due to lower suspicion of men with dry eye having underlying SS and deferred work-up which might play a role in increased mortality rate [15].

Multiple prior studies have reported a greater risk for malignancy in SS patients compared to the general population [16–18], particularly Non-Hodgkin’s Lymphoma (NHL) [6,19–22]. NHL, specifically mucosa-associated lymphoid tissue (MALT) origin, is thought to be due to a chronic inflammatory process related to chronic antigenic stimulation of B lymphocytes [23]. Not surprisingly, the presence of malignancy, specifically lymphoma, in patients with SS has been associated with worse clinical prognosis [7]. Current research has relied on systemic findings to identify variables, which may be predictive of long-term clinical outcomes in SS patients, such as the ESSDAI tool, which lacks the ocular manifestations of SS [6].

Recently there has been a heightened awareness among eye care professionals that patients with significant dry eye should be evaluated for underlying autoimmune conditions such as SS [24–26]. Although dry eye findings have been traditionally associated and emphasized in SS patients, 1 in 3 patients have other extraglandular ocular manifestations, that are less well-known [27]. Among these extraglandular ocular manifestations, certain complications can be vision-threatening, and have devastating outcomes if not appropriately treated. We previously reported that SS patients with significant extraglandular ocular complications have greater odds of also developing serious systemic complications [27]. However there are no studies to date which highlight the relationship between extraglandular ocular SS manifestations and mortality or systemic malignancy. The purpose of this retrospective study is to investigate the long-term consequences of significant (i.e. vision-threatening) ocular manifestations in patients with primary SS.

## Materials and methods

### Patients and medical evaluations

A study index was generated through the electronic medical records based on a diagnosis of “sicca syndrome” (International Classification of Diseases code 710.2) for all patients who were evaluated at Johns Hopkins Medical Institutions between January 2007 and May 2011 evaluated at the Jerome L. Greene Sjögren’s Syndrome Center at Johns Hopkins University School of Medicine in Baltimore, Maryland (earliest time period after implementing current research protocol). The diagnosis of SS was either established or confirmed at our institution. Patients were evaluated, treated, and followed in a multi-disciplinary fashion, but at least by ophthalmology and rheumatology specialties, throughout the follow-up period. The detailed description of the original data collection process and variables can be found elsewhere [27]. The study was approved by Johns Hopkins University Institutional Review Board. The study protocol adhered to the tenets of the Declaration of Helsinki and the Health Insurance Portability and Accountability Act. Written informed consent was obtained prior to patient enrollment.

### Clinical evaluation and diagnosis of Sjögren’s Syndrome

Systemic evaluations were tailored to the individual patient and included serological testing, minor salivary gland biopsy, salivary gland scintigraphy, parotid gland ultrasonography, and magnetic resonance imaging or computed tomography scanning of the major salivary glands. Ocular examinations included a full ophthalmologic exam by a single ophthalmologist (EKA). The presence of dry eye was assessed with tear film osmolarity, Schirmer test without topical anesthesia, tear film break-up time and ocular surface dye staining using lissamine green for conjunctiva and fluorescein for cornea, in the order listed here. Extraglandular ocular findings such as sterile keratolysis and scleritis were clinically diagnosed during slit lamp examination. Infectious etiology was ruled out through cultures or biopsies. A detailed description of the ocular and systemic examinations can be found elsewhere [27,28].

Serological markers of interest included: anti-Sjögren’s Syndrome A antibody (SS-A), anti-Sjögren’s Syndrome B antibody (SS-B), antinuclear antibody (ANA), rheumatoid factor (RF), erythrocyte sedimentation rate (ESR), and C-reactive protein (CRP). The most significant, or highest, ESR and CRP values recorded during an acute flare-up were considered. Other serological tests obtained according to clinical picture included antithyroid peroxidase antibody, antithyroglobulin antibody, immunoglobulins, IgG, C3 and C4. All serological tests were repeated at our institution to ensure uniformity and quality control. Orbital imaging was obtained to assess extraocular muscle thickening if clinically indicated, and conjunctival biopsy was performed for patients with significant chronic conjunctivitis with cicatrization [28].

The diagnosis of SS was made according to the American-European Consensus Group 2002 revised criteria [29] which requires the presence of at least 4 of 6 criteria, or 3 of 4 objective criteria, confirmed by two authors (SAR and ANB). *All* patients who were deemed to have SS had either positive anti-Sjögren’s Syndrome A or B antibody, or presence of positive minor salivary gland biopsy results. If the biopsy was performed elsewhere, the final report was obtained by one author (ANB) and reviewed. If there was any disparity, the glass slides were requested and reviewed prior to including the patient in the cohort. Historical account of SS was not sufficient to be included in this study.

### Data collection

The variables of interest included demographic information, serology, ocular and systemic findings on presentation and developed during the follow-up period. The data collection was performed in March 2019.

A chart review was performed to definitively determine the cause of death for the deceased patients by two trained researchers. When there were questions regarding the entries, one author (ANB) was the final arbiter, and a random sample of charts were reviewed by one author (EKA) to ensure the quality of the data. The data collection was complete with information from all available clinical visits (oldest record dating to October 1999) up to March 2019. All patients with less than three years of follow-up were excluded from the data analysis.

### Statistical analysis

Extraglandular ocular and systemic manifestations, occurring either at presentation or during follow-up, were compiled for all patients. Of the extraglandular ocular findings, sterile corneal ulcer/infiltration, corneal melt/perforation, cicatrizing conjunctivitis, uveitis, episcleritis/scleritis, optic neuropathy, and retinal vasculitis were considered to be “significant”. Systemic problems were carefully assessed and delineated as either direct manifestation of SS or unrelated co-morbidity. The ones considered direct manifestations of primary SS were: neurological findings, interstitial nephritis, vasculitis, autoimmune lung disease, primary biliary cholangitis, autoimmune hepatitis, autoimmune thyroid disease, erythema multiforme, and MALT lymphoma. Differences in proportion between any two binary variables (i.e. significant ocular finding, sex, malignancy, death) were compared using the chi-squared test. Fisher’s exact test was substituted for binary variables with inadequate sample size (n<5). Two-sided t-test was used to compare continuous variables between two groups (i.e. men/women, patients with and without extraglandular ocular findings). Odds ratios (OR) were calculated using binary logistic regression model using the Firth method (penalized likelihood) to reduce small-sample bias. P-values less than 0.05 were considered significant. Any patients with missing data was not included in the summary statistics or data analysis. Data analysis was performed with STATA version 14.2 (STATA Corp, College Station, Texas, USA).

## Results

Baseline characteristics of the cohort are displayed in Table 1. Of the 163 patients included in the original cohort [27], 126 patients (77.3%) had a regular follow-up of 3 years and were included in this analysis. Of note, many of the patients evaluated at the center came in for consultation only and were managed by their local physicians. The vast majority (92%) were women, and middle aged (49.7 years ± 13.0, no difference in age by sex). The proportion of men with SS was relatively small in this cohort. The ethnic distribution was representative of the patients referred to and treated at the Johns Hopkins Hospital, and predominantly Caucasian (82.5%). Two-thirds of the patients had a known diagnosis on presentation, and one-third (34.1%) were diagnosed with SS during the follow-up period. The duration of follow-up ranged from 3.0–15.9 years (mean 9.8 years, median 9.6 years, total of 1,235 patient-years). Nearly half of the patients (46.8%) had at least 10 years of follow-up data. Men were significantly less likely to be SS-A positive compared to women (p = 0.01), however there was no difference in SS-B positivity by sex (p = 0.30).

**Table 1:** Baseline characteristics in a longitudinal cohort of patients with primary Sjögren’s Syndrome evaluated at a multidisciplinary tertiary care center.

Characteristics	All Patients (n = 126)*
Women (%)	116 (92.1)
Ethnicity (%)	
White	104 (82.5)
Black	13 (10.3)
Hispanic	2 (1.6)
Asian	6 (4.8)
Other	1 (0.8)
Age at diagnosis of Sjögren’s syndrome (years)	
Mean (SD)	49.7 (13.0)
Duration of dry eye symptoms at presentation (years)	
Median	8.1
Mean (range)	10.4 (0.4–54.2)
Duration of follow-up (years)*	
Median	9.6 years
Mean (range)	9.8 years (3.0–15.9)
Baseline serology by sex**	
SS-A positive	
Men (n = 9)	3 (33.3%)
Women (n = 115)	88 (76.5%)
SS-B positive	
Men (n = 9)	2 (22.2%)
Women (n = 114)	51 (44.7%)

SD: Standard deviation.

*Fifty-nine patients (46.8%) had at least 10 years of follow-up data.

**Percentages determined by total of men or women with available serology.

The extraglandular ocular and systemic findings observed in the cohort are summarized in Table 2. One or more ocular manifestations were present in 37 patients (29.4%) either at presentation or during the follow-up period. Among these, significant extraglandular ocular manifestations occurred in 19 (15.1%) patients, and 3 of these patients had more than one significant ocular finding. One additional patient in the cohort developed a new significant ocular finding (concurrent anterior uveitis and scleritis) during the additional follow-up period since the previous study [27]. This was determined to be due to inadequate systemic immunosuppression and resolved with further treatment. Three of these 19 patients developed a new systemic extraglandular finding since the last analysis [27], including hepatocellular carcinoma in the setting of autoimmune hepatitis, MALT lymphoma, and SS-related inflammatory cardiac disease.

**Table 2:** Extraglandular ocular and systemic findings encountered in a longitudinal cohort of patients with primary Sjögren’s Syndrome evaluated at a multidisciplinary tertiary care center.*

Extraglandular Finding	N (%)
**OCULAR**	
Conjunctival	
Tarsal Conjunctiva	
Papillary or Follicular Conjunctivitis	15 (11.9)
Cicatrizing Conjunctivitis^Δ^	3 (2.4)
Bulbar Conjunctiva (chemosis)	2 (1.6)
Corneal	
Corneal Haze/Scarring	6 (4.8)
Sterile Corneal Ulcer/Melt/Perforation^Δ^	8 (6.3)
Episcleritis/Scleritis^Δ^	4 (3.2)
Uveitis^Δ^	4 (3.2)
Retinal vasculitis^Δ^	1 (0.8)
Optic Neuropathy/Neuritis^Δ^	2 (1.6)
Orbital Inflammation	3 (2.4)
**SYSTEMIC**	
Skin (psoriasis, erythema multiforme)	3 (2.4)
Neurological Findings	37 (29.4)
Vasculitis	9 (7.1)
Visceral Organ Involvement	
Interstitial Nephritis	5 (4.0)
Autoimmune Lung Disease	4 (3.2)
Autoimmune Cardiac Disease	1 (0.8)
Gastrointestinal Disease^+^	8 (6.3)
Thyroid Disease^◆^	31 (24.6)
Hematological Abnormalities	3 (2.4)
Malignancy	19 (15.1)
Hematologic Malignancies	6 (4.8)
MALT Lymphoma	5 (4.0)
Mantle Cell Lymphoma	1 (0.8)
Solid Organ Malignancies	13 (10.3)

N = Number of patients with the finding.

*Median years of follow-up = 9.6 years (range 3.0–15.9). Includes findings at initial presentation and those which developed during follow-up.

^Δ^Classified as ‘significant’ extraglandular ocular finding.

^+^Including Inflammatory Bowel Disease and Autoimmune Hepatitis.

^◆^Including Hashimoto’s/Grave’s Disease and thyroid disease of unspecified etiology.

MALT: Mucosa-associated lymphoid tissue.

The most common systemic findings were neurologic (29.4%) and thyroid (24.6%) disease, followed by malignancy (15.1%) (Table 2). Patients with significant ocular manifestations were 3.6 times more likely to have extraglandular systemic SS manifestations (95% confidence interval [CI], 1.3–10.6; p = 0.02) when adjusted for age at diagnosis, similar to our previous report (OR = 3.9) [27].

The serology of the cohort is detailed in Table 3. The positivity rate for SS-A, SS-B, ANA, RF, and anti-CCP did not differ in patients with significant extraglandular ocular findings versus those without (p>0.05 for all), however they had significantly higher mean ESR and CRP (p = 0.001 and p = 0.003, respectively).

**Table 3:** Serological findings in a longitudinal cohort of patients with primary Sjögren’s Syndrome evaluated at a multidisciplinary tertiary care center.*

	Patients with Significant Extraglandular Ocular Findings N = 19	All patients N = 126	p-value**
SS-A positive	12 (66.7%)	91 (73.4%)	0.49
SS-B positive	7 (38.9%)	53 (43.1%)	0.70
ANA positive	15 (83.3%)	111 (90.2%)	0.38
High titer ANA (1:320 or greater)	9 (64.3%)	86 (80.4%)	0.14
RF positive	8 (50%)	73 (59.4%)	0.41
Anti-CCP	2 (28.5%)	9 (12.7%)	0.21
ESR, mean (SD)	56.1 (42.9)	33.4 (27.7)	**0.001**
CRP, mean (SD)	8.5 (6.2)	2.0 (8.9)	**0.003**

*Data indicates number (%) unless otherwise indicated. Percentage determined by the total number of patients with available serology results for each specific test.

**P-value refers to difference in proportion (or mean for continuous variables) for each serological marker in patients with significant extraglandular ocular findings versus those without. **Bolded** values indicate statistical significance, p<0.05.

SS-A: Anti-Sjögren’s Syndrome A antibody; SS-B: Anti-Sjögren’s Syndrome B antibody; ANA: Antinuclear antibody; RF: Rheumatoid factor; CCP: Anti-cyclic citrullinated peptide; ESR: Erythrocyte sedimentation rate, CRP: C-reactive protein; SD: Standard deviation.

The characteristics of patients having malignancy either at presentation or during follow-up are summarized in Table 4. A total of 19 patients (15.1%) had a hematologic or solid malignancy. The most common hematologic malignancy was NHL. The lifetime prevalence of NHL in this cohort was 4.8% (median follow-up 9.8 years), almost double the estimate from our initial study with a shorter follow-up (2.7%, median follow-up 3.0 years) [27]. The most common solid malignancy was breast cancer (6.0%). Patients with extraglandular systemic findings tended to have concomitant or history of malignancy compared to those without systemic findings (22.0% vs 10.5%, p = 0.07), although this was not statistically significant. Lastly, a greater proportion of men had a history of or concurrent malignancy compared to women (30.0% versus 13.7%, p = 0.16), although this difference did not reach statistical significance.

**Table 4:** Concomitant hematologic or solid organ malignancies in a longitudinal cohort of patients with primary Sjögren’s Syndrome evaluated at a multidisciplinary tertiary care center.

	N (%) N = 126
Total	19 (15.1)
Hematologic Malignancy	
Non-Hodgkin’s Lymphoma	6 (4.8)
Solid Malignancy	
Breast*	7/116 (6.0)
Thyroid	2 (1.6)
Hepatocellular	1 (0.8)
Lung	1 (0.8)
Pancreatic	1 (0.8)
Prostate*	1/10 (10.0)

*Denominator for prevalence of breast cancer = number of total women; denominator for prevalence of prostate cancer = number of total men.

Ten of the 19 patients with significant extraglandular ocular manifestations initially presented with inflammatory keratolysis (including corneal ulcer, melt, and/or perforation) or scleritis. There was a significantly higher proportion of men with inflammatory keratolysis or scleritis compared to women (40% versus 5.2%, p = 0.003). Also, these patients were less likely to be SS-A positive (p = 0.05), however this finding was not significant when adjusted by sex (p = 0.20). They had a higher prevalence of anti-cyclic citrullinated peptide (anti-CCP) antibodies, even when adjusted for sex, although this was not statistically significant (p = 0.07). Lastly, these 10 patients had a significantly higher mean ESR compared to the rest of the cohort (64.8 vs 21.9, p = 0.009).

A total of five patients died (5/126, 5.0%) during the follow-up period. Two of these patients had a history of significant ocular manifestations. They initially presented with sterile corneal melt/perforation (n = 1) and scleritis (n = 1), were appropriately treated with systemic medications, and had no recurrence of their ocular findings during follow-up. However, they died from complications of their underlying SS disease (MALT lymphoma and SS related cardiac disease). Patients with inflammatory keratolysis or scleritis had a 2.3-fold greater incidence of death compared to the rest of the cohort (OR = 2.3, 95% confidence interval [CI] 0.5 to 4.0, p = 0.01). The other three patients (without a history of significant ocular manifestations) died from chronic conditions unrelated to their SS diagnosis (unstable angina with end stage renal disease, myelodysplastic syndrome, and breast cancer). Lastly, significantly higher mortality was observed in men (2/10, 20.0%) compared to women (3/116, 2.6%) (p = 0.05) in the cohort.

## Discussion

This retrospective study aimed to assess the longer-term morbidity and mortality of a longitudinal cohort of primary SS patients previously studied [27]. Approximately 15.1% of the cohort had a history of concomitant malignancy, with 4.8% having NHL. Patients with inflammatory keratolysis or scleritis had a 2.3 times greater incidence of death, either from malignancy or direct results of SS complications, compared to the rest of the cohort. Lastly, men patients with SS had a higher incidence of death compared to women.

There have not been any studies to date assessing the consequences of significant extraglandular ocular manifestations of SS. Several studies have observed that patients with extraglandular *systemic* manifestations, such as vasculitis, have higher mortality compared to patients without systemic involvement [7,10,30]. Unfortunately, none of these reports studied the significance of *extraglandular ocular* findings. We found a higher incidence of death in patients with significant extraglandular ocular findings. Patients specifically with inflammatory keratolysis or scleritis had double the mortality compared to the rest of the cohort. We propose that perhaps the most serious extraglandular ocular manifestations of primary SS is inflammatory keratolysis and scleritis, which could represent the highest degree of systemic disease activity. Indeed, these patients had significantly higher ESR and CRP.

The ESSDAI score, intended to quantify SS disease activity has previously been linked to a higher risk of death [31]. The ESSDAI score represents twelve organ systems, but does not include any of the ocular manifestations [9]. Our study suggests that significant ocular complications are related to higher incidence of death, and therefore likely related to the overall systemic disease activity. Therefore, the ESSDAI should include significant ocular manifestations as an additional organ system.

Several population-based studies have reported a higher rate of malignancy in patients with primary SS, ranging between 11.5%-28.3% [18,32–34]. The prevalence of malignancy (either history of or concomitant) in our cohort was 15.1%, falling within this range. The most common hematologic malignancy was NHL, which occurred in 4.8% of the entire cohort, almost double what was previously reported in this same cohort [27]. This increase is likely attributable to the considerably longer follow-up and consistent with the prior studies [19,23,30,34,35].

The most common malignancy in our cohort was breast cancer, occurring in 6.0% of women, either in the past or concurrently. A recent report from Argentina also found a higher overall incidence of breast cancer compared to the general population (2.5%), although this study included only new cases during follow-up [18]. Previous research has demonstrated that specific clinical findings (vasculitis) and high scores with quantitative assessments (ESSDAI) are associated with malignancy [30,34,36]. In our study too we found that patients with extraglandular systemic findings, but not extraglandular ocular findings, were more likely to have malignancy. This is likely because of insufficient number of patients in our cohort to demonstrate statistical significance. Nevertheless, significant extraglandular ocular manifestations should be considered when assessing disease severity at presentation, as this could play a role in predicting a patient’s future prognosis [9].

Additionally, our study further confirmed our previous findings that SS is more severe and devastating in men [15]. The men in this study had a higher incidence of death and malignancy compared to women. Even though only 8% of the patients were men, almost half of the inflammatory keratolysis or scleritis cases occurred in men. Interestingly, they were all SS-A seronegative. Thus clinicians should be especially cognizant when evaluating male patients with clinically significant dry eye to avoid missing a diagnosis of underlying SS with possible morbidity and mortality.

Similar to our previous study [27], we confirmed that patients with significant extraglandular ocular findings were more likely to have systemic involvement (odds ratio = 3.6). In addition, we found that patients with significant ocular findings had significantly higher systemic inflammatory markers (ESR and CRP) indicating greater disease activity. A greater proportion of patients with inflammatory keratolysis or scleritis also had anti-CCP positivity. It is possible that these patients have a more complex pathophysiology or “polyautoimmunity” [37,38].

Ultimately, these significant extraglandular ocular findings should be considered equivalent to other serious systemic complications of SS. Scleritis is vasculitis of the eye [39], and therefore should be treated swiftly and appropriately with similar immunosuppressive agents used in other vasculitides to avoid future morbidity and mortality. However central or paracentral corneal ulceration/melts is different, and not generally related to inflammation of the limbal vessels [28,40]. The inflammatory pathway of sterile keratolysis [41] appears to be more similar to fibrotic complications of SS disease such as interstitial lung disease, and should be treated with appropriate systemic immunosuppressive medications as well. Understanding the pathophysiology of these significant extraglandular ocular complications of SS is important for determining appropriate systemic treatment as well as clinical prognosis.

There are several limitations of this study. The study cohort includes patients seen at a tertiary referral center and may not represent the general SS population. Due to the retrospective nature of the analysis, we can only discuss associations between variables and not causal relationships. With regards to the mortality analysis, it is possible that other patients died and the hospital medical records were not updated. However, efforts were made to contact patients if they missed their regularly scheduled visits. Additionally, due to the limited amount of data, patients, and rare event occurrences (i.e. mortality), we were unable to perform multivariable analyses which may have provided more insight regarding the relationship between extraglandular findings and clinical outcomes. The results should be interpreted cautiously in the setting of small sample size. Nonetheless we hope that our findings serve an important purpose with establishing the potential role and prognostic value of extraglandular ocular manifestations.

Primary SS is estimated to affect 2 to 4 million Americans [42], which is actually greater than the number of individuals with rheumatoid arthritis [43], and therefore might as well be the most common autoimmune disease. Unfortunately, primary SS still remains underdiagnosed and ocular findings are underappreciated despite the devastating consequences. Eye care providers are in a unique position to carefully screen all dry eye patients for possible underlying disease, particularly SS, so that they can be appropriately identified and treated.
